# Intestinal colonization with *Candida auris* and mucosal immune response in mice treated with cefoperazone oral antibiotic

**DOI:** 10.3389/fimmu.2023.1123200

**Published:** 2023-04-11

**Authors:** Diprasom Das, Harm HogenEsch, Shankar Thangamani

**Affiliations:** ^1^ Department of Comparative Pathobiology, College of Veterinary Medicine, Purdue University, West Lafayette, IN, United States; ^2^ Purdue Institute for Immunology, Inflammation and Infectious Diseases (PI4D), West Lafayette, IN, United States

**Keywords:** *Candida auris*, antibiotic treatment, intestinal colonization, dissemination, mucosal immunity, Antifungal therapeutics

## Abstract

*Candida auris*, an emerging multi-drug resistant fungal pathogen, causes invasive infections in humans. The factors regulating the colonization of *C. auris* in host niches are not well understood. In this study, we examined the effect of antibiotic-induced gut dysbiosis on *C. auris* intestinal colonization, dissemination, microbiome composition and the mucosal immune response. Our results indicate that mice treated with cefoperazone alone had a significant increase in *C. auris* intestinal colonization compared to untreated control groups. A significant increase in the dissemination of *C. auris* from the intestine to internal organs was observed in antibiotic-treated immunosuppressed mice. Intestinal colonization of *C. auris* alters the microbiome composition of antibiotic-treated mice. Relative abundance of firmicutes members mainly *Clostridiales* and *Paenibacillus* were considerably increased in the cefoperazone-treated mice infected with *C. auris* compared to cefoperazone-treated uninfected mice. Next, we examined the mucosal immune response of *C. auris* infected mice and compared the results with *Candida albicans* infection. The number of CD11b+ CX3CR1+ macrophages was significantly decreased in the intestine of *C. auris* infected mice when compared to *C. albicans* infection. On the other hand, both *C. auris* and *C. albicans* infected mice had a comparable increase of the number of Th17 and Th22 cells in the intestine. A significant increase in *Candida*-specific IgA was observed in the serum of *C. auris* but not in the *C. albicans* infected mice. Taken together, treatment with broad-spectrum antibiotic increased the colonization and dissemination of *C. auris* from the intestine. Furthermore, findings from this study for the first time revealed the microbiome composition, innate and adaptive cellular immune response to intestinal infection with *C. auris*.

## Introduction

Fungal infections such as invasive candidiasis affect about 700,000 patients globally and result in high mortality rate of more than 40% among infected patients (1–3). Furthermore, indiscriminate use of anti-microbial drugs in COVID-19 patients resulted in the emergence of multidrug-resistant fungal pathogens such as *Candida auris* (4, 5)*. C. auris* was recently classified as an urgent threat by the US Centers for Disease Control and Prevention (CDC) Antibiotic Threats Report (2019) and ranked in the critical priority group by the World Health Organization (WHO) in a recently released list of fungal priority pathogens (6–8). Furthermore, *C. auris* strains exhibit resistance to FDA-approved antifungal drugs partly through mutation in the target genes (1, 2, 4, 6). *C. auris* colonizes the human skin resulting in nosocomial transmission and outbreaks of invasive infections (5). Emerging evidence from recent clinical studies indicate that individuals on prior antibiotic therapy (30-45%) were highly susceptible to *C. auris* infections (3–5, 9). Furthermore, *C*. *auris* was detected in human stool samples, indicating that the intestine is a potential colonization site (10–12). However, the effect of antibiotic treatment on intestinal colonization of *C. auris* is not clear.

Antibiotic-induced gut dysbiosis and dysregulation in host defense increases the susceptibility to *Candida albicans* colonization and invasion from the intestine (13–19). Invasive *Candida* infections originating from the initial colonization of the intestine and subsequent dissemination results in fatal infections in immunocompromised patients (20–22). Therefore, understanding the factors regulating *Candida* intestinal colonization and mucosal antifungal defense is critical to develop novel therapeutic approaches to prevent and treat *Candida* infections (14, 20–25). In this study, we investigated the effect of cefoperazone, a widely used FDA-approved broad-spectrum antibiotic, on *C. auris* intestinal colonization and mucosal immune defense against *C. auris.* Our findings suggest that treatment with broad-spectrum antibiotic alone increases the susceptibility to *C. auris* colonization, dissemination and alters the microbiome composition in the intestine. We examined the cellular and adaptive immune response in the intestine of *C. auris* infected mice and compared with *C. albicans* infected groups. Our findings are significant as they show that oral broad-spectrum antibiotic treatment is a major risk factor for intestinal colonization of *C. auris* and subsequent invasive infections.

## Materials and methods

### Strains and reagents.

*Candida albicans* SC5314 was provided by Dr. Andrew Koh (University of Texas Southwestern Medical Center, USA). *Candida auris* #0387 (South Asian strain) was obtained from Center for Disease Control (CDC) AR Isolate Bank, USA. The reagents and chemicals for all the experiments were purchased from the indicated vendors mentioned below. Yeast Peptone Dextrose (YPD) (242810, BD Difco, Franklin Lakes, NJ, USA), CHROMagar (CA222, CHROMagar, Paris, France), agar (BP1423-500, Fisher Bioreagents, Pittsburg, PA, USA), ampicillin (14417, Cayman Chemicals, Ann Arbor, MI, USA), kanamycin-Sulfate (BP9069-5, Fisher Bioreagents, Pittsburg), formaldehyde (F79, Fisher Bioreagents, Pittsburg) and streptomycin (100556, MP Biomedicals, Santa Ana, CA, USA), cefoperazone (Sodium salt) (16113, Cayman Chemical), cyclophosphamide (PHR1404, Millipore Sigma, St. Louis, MO, USA), U-bottom 96 well plate (229190) from Cell Treat (Pepperell, MA, USA). RPMI-1640 (SH30027.02, Cytiva, Marlborough, MA, USA), HEPES (BP310-500, Fisher Bioreagents), fetal bovine serum (FBS-500-H, CPS Serum, Parkville, MO, USA), penicillin-streptomycin (P4333-100ML, Millipore Sigma), L-glutamine (G3126, Millipore Sigma), gentamycin (15710-072 Life Technologies, Carlsbad, CA, USA), D-glucose (D16-500, Fisher Bioreagents), 0.5 M EDTA (15575-020, Life Technologies), MgCl_2_ hexahydrate (M2670, Millipore Sigma), CaCl_2_ hexahydrate (21108, Millipore Sigma), 10× Hanks Balanced Salt Solution (HBSS) (20-021-CV, Corning, Corning, NY, USA), Percoll (GE17-0891-01, Millipore Sigma), 70 μm cell strainer (22-363-548, Fisher Scientific), collagenase Type 1 (17100-017, GIBCO). For immune cell staining, anti-mouse antibodies were purchased from BioLegend, San Diego, CA, USA. Zombie Violet Dye, CD16/32 (Clone 93), CD3ϵ (FITC, 145-2C11), CD4(APC, GK1.5), Ly-6G (PE/Cyanine7, 1A8), CD11b (PE, M1/70), F4/80 (Alexa Fluor 700, BM8), CX3CR1(Alexa Fluor 488, SA011F11), IFNγ (Alexa Fluor 700, XMG1.2), IL17A (PE/Dazzle 594, TC11-18H10.1), IL5(PE, TRFK5). IL22 (APC, IL22JOP) anti-mouse antibody was purchased from Invitrogen, Waltham, MA, USA. The cell staining buffer, cell activation cocktail without brefeldin A(500X), monensin (1000X), intracellular staining permeable wash buffer (10X) and fixation wash buffer were purchased from BioLegend, San Diego, CA, USA. IgA mouse uncoated ELISA (enzyme-linked immunoassay) kit (88-50450), IgG1mouse uncoated ELISA kit (88-50410), heparinized capillary tubes (22-260-950), ELISA wash buffer (00-0400-59), Nunc MaxiSorp flat bottom 96 well plate (50-112-3685) was purchased from Invitrogen, Waltham, MA, USA

### Cefoperazone treatment and fungal infection in mice

All animal studies were approved by the Institutional Animal Care and Use Committee at Purdue University. Sterile water supplemented with or without cefoperazone (0.5mg/ml) was given to 8-10 weeks old male and female C57BL/6J mice as described previously (17, 26). Cefoperazone-supplemented water was changed every 3 days till the end of the experiment. After 7 days of antibiotic treatment, mice were infected with *C. auris* CDC #0387 (or) *C. albicans* SC314 via oral gavage at a dose of about ~1 x 10^8^ CFU (Colony Forming Units) per mouse. Fecal pellets were collected from individual mice from both antibiotic-treated and untreated groups on indicated days to determine the fungal load. At 12 days post infection, mice from both untreated and antibiotic-treatment group were euthanized and gut contents were collected. Gut contents were weighed and homogenized using 1× PBS and the supernatant was plated onto YPD-agar plates containing 100 μg/ml ampicillin, 50 μg/ml kanamycin, and 100 μg/ml streptomycin. *C. auris* and *C. albicans* colonies were confirmed by plating the supernatants onto ChromAgar plates. Plates were incubated at 37°C for 24 hours to determine the fungal load (CFU/gram of fecal matter).

### *C. auris* dissemination in immunosuppressed mouse model

The dissemination potential of *C. auris* CDC #0387 was determined in cyclophosphamide-treated mice as described before (16). Briefly, mice treated with or without cefoperazone for 7 days were infected with *C. auris*. Three days post-infection, all mice were treated with 150 mg of cyclophosphamide per kilogram of mouse body weight by intraperitoneal injection. A second and third dose of cyclophosphamide was given at 5- and 7-days post-infection, respectively. Five days after the last dose of cyclophosphamide, the mice were euthanized to determine the fungal load in cecal contents, liver, and kidneys as described before (16).

### Isolation and staining of immune cells from intestinal lamina propria (LP)

Mononuclear cells from large intestinal LP were isolated as described previously (27). Briefly, intestine removed from mice was kept in cold RPMI harvest media (RPMI-1640, 5% FBS (fetal bovine serum), 10mM D-Glucose, 4 mM sodium bicarbonate, 5mM HEPES, 2mM L-glutamine, 20 U/ml penicillin, 20µg/ml gentamycin) to remove mesenteric fat and fecal material. Intestinal pieces were washed three times with EDTA solution (1X Hanks Balanced Salt Solution (HBSS), 5mM HEPES, 2mM L-glutamine, 20 U/ml penicillin, 20µg/ml gentamycin, 1.3 mM EDTA, water,4 mM sodium bicarbonate) and digested with collagenase solution (100U/ml Type I collagenase, RPMI-1640, 5% FBS, 5mM HEPES, 2mM L-glutamine, 20 U/ml penicillin, 20µg/ml gentamycin, 1mM magnesium chloride, 1mM calcium chloride). The resulting digest was strained through a 70-micron cell strainer and mononuclear strains obtained were used for staining. Cell labeling was performed in a U-bottom 96 well plate. For surface staining, cells were washed with cell staining buffer and Fc receptors were blocked with anti-mouse CD16/32. Respective antibodies for surface markers were used at a 1:100 dilution. After surface staining, cells were fixed using fixation buffer. For intracellular cytokine staining, cells were stimulated for 4.5 hours with cell activation cocktail at 37°C at 5% CO_2_ according to the manufacturer’s instructions. Cells were next incubated with 1X intracellular staining perm wash buffer with indicated antibodies. Cells were washed and the flow cytometry data was acquired with a Attune NxT Flow Cytometer (Invitrogen, Carlsbad, CA) and analyzed using FlowJo software (Eugene, OR).

### Microbiome analysis

Groups of mice treated with either sterile water or cefoperazone-containing water for 7 days and infected with (or) without *C. auris* CDC #0387 strain. Antibiotic treatment was continued till the end of the experiment. After 10 days of infection, mice were euthanized and cecal contents were collected to analyze the microbiome composition using MiSeq Illumina platform as described before (17). Microbiome sequences were analyzed using Qiime2 and the sequences were aligned by mafft and a phylogenetic tree was constructed using fasttree as before (17).

### ELISA to detect total and fungal specific IgA and IgG1

Mice pre-treated with cefoperazone and infected with *C. auris* or *C. albicans* were euthanized 10 days post-infection and the gut contents collected were used to measure the antibodies level. Uninfected antibiotic-treated mice were used as control. Blood samples collected from these mice through retro-orbital route were used to measure IgA and IgG1 level in the serum. Gut contents were resuspended in sterile 1X PBS to a concentration of 250 mg/ml. Samples were homogenized for 1 minute by vortexing and the homogenates were spun at 10,000g for 10 min and the supernatants were used for ELISA. Total IgA was quantified by diluting small intestine and serum samples to 1: 1000 and large intestine samples to 1: 100. Total IgG1 was quantified by diluting small intestine and large intestine samples to 1: 10 and serum samples to 1: 100. Total IgA and IgG1 were determined as per the manufacturer’s instructions (IgA, catalogue #88-50450 and IgG1, Catalogue#88-50410 from Invitrogen). To determine fungal specific IgA and IgG1 levels, *C. albicans* or *C. auris* lysates were prepared and ELISA was carried out as described elsewhere (18). Briefly, overnight culture of *C. albicans* and *C. auris* were fixed in 4% paraformaldehyde for an hour at 4°C. Then fungal suspensions were washed three times in molecular-grade water by centrifuging at 900 x g for 2 minutes and were freeze-thawed three times cycle of 10 minutes in dry ice and 75°C heat block. The suspensions were then sonicated for 8 cycles of 15 seconds on/30 seconds off and the debris were centrifuged for 4000 rpm for 10 minutes. The supernatant collected was diluted to 1:50 in 1X PBS (coating buffer) and plates were coated for overnight at 4°C. To quantify fungal-specific IgA and IgG1, contents from large and small intestine, and serum samples were diluted to 1: 4. Next day, 96-well plates were washed 3 times with wash buffer (catalogue# 00-0400-59, Invitrogen) and the wells were blocked by adding 250μl of PBS containing 0.1% tween 20 and 1% BSA at room temperature (RT) for 2 hours. To detect IgG1 level, wells were washed two times after incubation and then 50μl of diluted samples and 50μl of PBS containing 0.1% tween 20 and 1% BSA were added. Then 50μl of detection antibody (horseradish peroxidase (HRP)-conjugated anti-mouse IgG polyclonal antibody, catalogue #88-50410, Invitrogen) was added and the plate was incubated for 2 hours at RT. To detect IgA level, 10μl of diluted samples and 90μl of PBS containing 0.1% tween 20 and 1% BSA were added to each well and incubated for 2 hours at RT. After incubation, wells were washed and 100μl of detection antibody (HRP-conjugated anti-mouse IgA polyclonal antibody, catalogue#88-50450, Invitrogen) was added to each well and incubated for one hour at RT. Then wells are washed for the final time and 100μl of tetramethylbenzidine (TMB) substrate solution was added to each well and incubated for 10-15 minutes at RT. The reaction was stopped by adding 2N H_2_SO_4_ and absorbance was measured at 450 nm to determine the O.D by spectrophotometer.

### Statistics analysis

Statistical analysis was done using Mann-Whitney U test and Kruskal-Wallis test followed by Dunn’s multiple comparison test using GraphPad Prism 9.2.0 (GraphPad Software, La Jolla, CA). *P* values of ≤ 0.05 were considered significant.

## Results

### Cefoperazone treatment increases the intestinal colonization of *C auris* in mice

**Figure 1:**
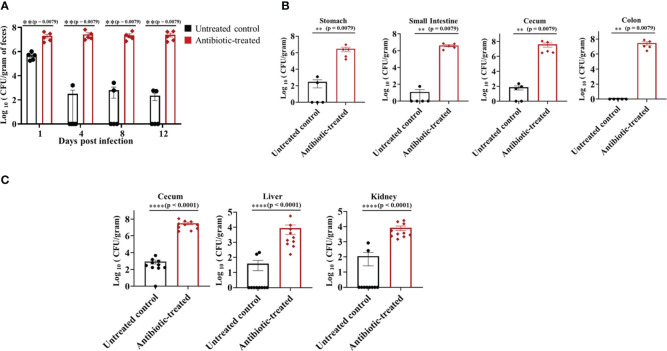
Oral antibiotic treatment increased *C. auris* colonization in the gastrointestinal tract of mice. Groups of mice were pre-treated with either sterile drinking water (untreated control group) or cefoperazone for 7 days and infected with *C. auris* 0387 strain via oral gavage. Antibiotic-treatment was continued till the end of the experiment. **(A)** Fungal load in the fecal pellets collected on indicated days and colony forming units (CFU) were determined by plating the contents onto YPD agar containing antibiotics (ampicillin, kanamycin, and streptomycin). *C. auris* colonies were further confirmed by plating onto chromagar plates. **(B)** After 12 days, mice were euthanized and gut contents collected were used to determine the fungal load in stomach, small intestine, cecum and colon. Data are presented as mean ± SEM with n = 5 mice for each group. **(C)** Untreated and antibiotic-treated mice were infected with *C. auris* and treated with cyclophosphamide. After 5 days of infection, mice were euthanized to determine the fungal load in cecal contents, liver and kidney. The bars indicate the mean ± SEM with n = 10 mice for each group. Statistical significance was calculated using Mann-Whitney U test. **p ≤ 0.01; ****p ≤ 0.0001.

Groups of mice treated with sterile water or water containing cefoperazone were infected with *C. auris* CDC #0387 (South Asian strain) via oral gavage at a dose of ~1 × 10^8^ colony forming units (CFU) per mouse. Antibiotic-treatment was continued throughout the experiment. Fecal pellets were collected from untreated control and antibiotic-treated groups on days 1, 4, 8 and 12 post-infection to determine the fungal load. The antibiotic-treated mice showed significantly higher *C. auris* load in fecal samples compared to untreated control groups from 1 to 12 days post-infection (**Figure 1A**). The fungal load was increased by almost 2 log_10_ on Day 1 and by more than 4 log_10_ in the fecal samples of antibiotic-treated mice. *C. auris* was undetectable in more than 50% of mice from the untreated control group (**Figure 1A**). After 12 days, mice were euthanized to determine the fungal load in gut contents including stomach, small intestine, cecum and colon. Gut contents from antibiotic-treated mice had a significantly higher (4-5 log_10_) fungal load compared to untreated control group (**Figure 1B**). Average fungal load in stomach and small intestine of antibiotic treated mice was 2.5- 5 x 10^6^ CFU/gram of contents, whereas cecum and colon had 2.5 – 5 x 10^7^ CFU/gram of contents (**Figure 1B**). On the other hand, fungal loads in the stomach, small intestine and cecum of untreated control groups were about 10^1^- 10^2^ CFU/gram of contents. No detectable level of *C. auris* was observed in more than 50% of gut contents from untreated control mice. Collectively, our findings indicate that treatment with a broad-spectrum antibiotic increased colonization of *C. auris* in the gastrointestinal tract.

### *C. auris* disseminate from the intestine of cefoperazone treated immunocompromised mice

To examine if *C. auris* disseminates from the intestine of immunocompromised mice to cause a systemic infection, mice treated with and without cefoperazone were infected with *C. auris* and treated with cyclophosphamide. After 5 days of infection, fungal loads in cecum, liver and kidney were determined. The fungal load was significantly increased in cecal contents, liver and kidney of the antibiotic-treated group compared to control mice that received water (**Figure 1C**). The fungal load in cecal content, liver and kidney of antibiotic-treated mice was about 10^7^, 10^4^ and 10^4^ CFUs/gram compared with 10^2^-10^3^ CFUs/gram in control mice. Fungal organisms were undetectable in liver and kidney of eight control mice. Collectively, these results indicate a significant increase in dissemination of *C. auris* from the intestine to internal organs in the antibiotic-treated mice compared with untreated control groups following immunosuppressive treatment.

### Intestinal colonization of *C. auris* in the antibiotic-treated mice alters the microbiome composition

**Figure 2:**
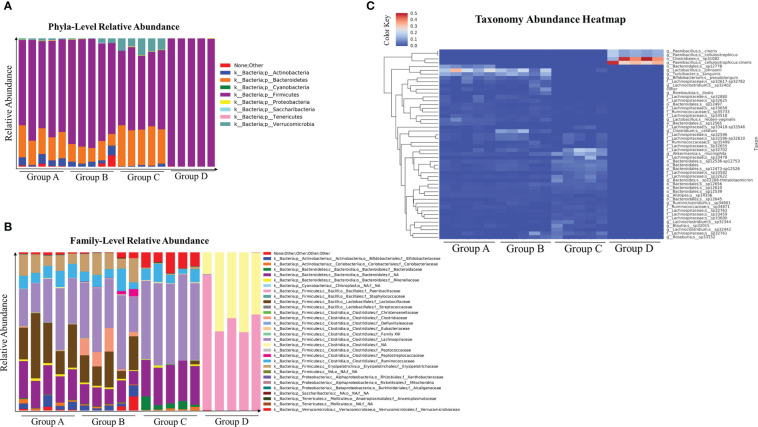
Microbial composition at taxonomical level and abundance heatmap of gut microbial community in the cecal contents of uninfected and *C. auris* infected mice were shown here. Groups of mice treated with sterile water or cefoperazone containing water for 7 days were infected with and without *C. auris*. Group A (received sterile water without *C. auris* infection), Group B (received sterile water with *C. auris* infection), Group C (received antibiotic water without *C. auris* infection), and Group D (received antibiotic water with *C. auris* infection). Relative abundance of major phyla **(A)** and family level abundance **(B)**, taxonomical abundance heatmap with top fifty abundant microbial species **(C)** among all four groups were plotted. The relative abundance score ranges from 0 to 0.5. Five mice per group was used for microbiome analysis.

Antibiotic treated mice infected with *C. albicans* alters the composition of gut microbiome (17). Therefore, we examined the microbiome composition in the cefoperazone-treated mice infected with *C. auris*. Our results indicate there is no considerable change in the microbiome composition in mice that received sterile water and infected with or without *C. auris* (**Figures 2A-C**). However, we observed considerable alteration in the microbiome composition between antibiotic-treated mice infected with and without *C. auris* (**Figures 2A-C**). Relative abundance of firmicutes members mainly *Clostridiales* and *Paenibacillus* were considerably increased in the cefoperazone-treated mice infected with *C. auris* compared to cefoperazone-treated uninfected mice (**Figures 2A-C**). Collectively, our results indicate that intestinal colonization of *C. auris* in the antibiotic-treated mice alters the microbiome composition.

### Innate immune response in the intestine of *C. auris* and *C. albicans*-infected antibiotic treated mice

**Figure 3:**
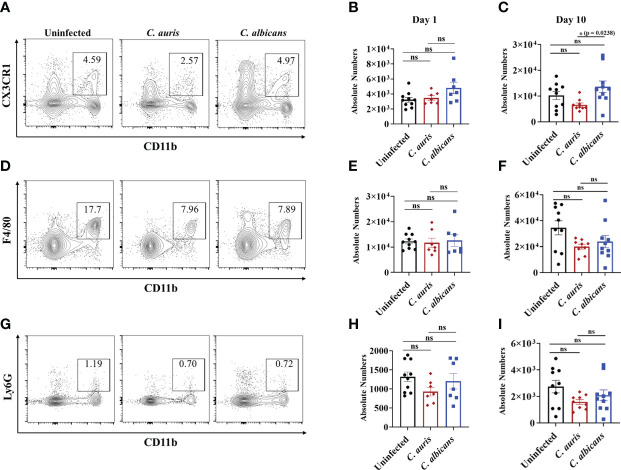
Innate immune response in the intestine of *C. auris* and *C. albicans* infected mice groups. Mice treated with cefoperazone for 7 days and infected with *C. auris* 0387 (or) *C. albicans* SC5314. Antibiotic treatment was continued till the end of experiment and groups of uninfected mice were used as control. **(A, D, G)** Representative flow plots of CD11b+ CX3CR1+ macrophages, CD11b+ F4/80+ macrophages and CD11b+ Ly6G+ neutrophils from the large intestine of uninfected, *C. auris* (or) *C. albicans* infected mice after 10-12 days post infection was shown here. **(B, E, H)** Absolute number of CD11b+ CX3CR1+ macrophages, CD11b+ F4/80+ macrophages and CD11b+ Ly6G+ neutrophils from the large intestine of uninfected, *C. auris* (or) *C. albicans* infected mice after one day post infection was shown here. **(C, F, I)** Absolute number of CD11b+ CX3CR1+ macrophages, CD11b+ F4/80+ macrophages and CD11b+ Ly6G+ neutrophils from the large intestine of uninfected, *C. auris* (or) *C. albicans* infected mice after 10-12 days post infection was shown here. Each group had 7-10 mice and the data represents mean ± SEM. Statistical significance of differences between the groups was calculated using the Kruskal-Wallis test followed by Dunn’s multiple comparison test with ns – nonsignificant, *p ≤ 0.05.

Innate immune cells such as macrophages and neutrophils play a critical role in host defense against *C. albicans* in the intestine (13, 28, 29). Furthermore, a recent study indicates that *C. auris* elicits less immunoinflammatory response than *C. albicans* (30). Therefore, we used groups of mice treated with cefoperazone and infected orally with *C. auris* or *C. albicans* to compare the innate immune cells between these two *Candida* species. Uninfected mice were used as a control. We examined three major innate immune cells; CD11b+ CX3CR1+ macrophages, CD11b+ F4/80+ macrophages and CD11b+ Ly-6G+ neutrophils in the large intestine of infected and uninfected groups. At day 1 post-infection, there was no significant change in the absolute cell number of three innate immune cells among all three groups (**Figures 3B, E, H**). After 10 days of infection, the absolute number of CD11b+ CX3CR1+ macrophages was significantly decreased in the intestine of *C. auris* infected mice compared to *C. albicans-*infected groups (**Figures 3A, C**). No significant change in the absolute number of CD11b+ F4/80+ macrophages (**Figures 3D, F**) and CD11b+ Ly-6G+ neutrophils (**Figures 3G, I**) were observed among all three groups. Taken together, decreased number of CD11b+ CX3CR1+ macrophages were observed in the intestine of *C. auris*-infected mice compared to *C. albicans*- infected groups.

### Adaptive immune response in the *C. auris* and *C. albicans* infected antibiotic treated mice

**Figure 4:**
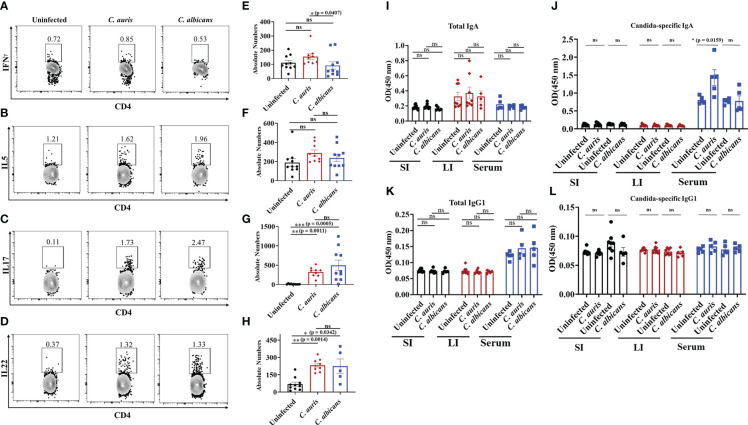
Adaptive immune response in the intestine of *C. auris* and *C. albicans* infected mice groups. Mice treated with cefoperazone for 7 days and infected with *C. auris* 0387 (or) *C. albicans* SC5314. Antibiotic treatment was continued till the end of experiment and groups of uninfected mice were used as control. After 10-12 days of infection, mice were euthanized to isolate and stain monocular cells from large intestine. Gut contents and serum were collected to assess the total and Candida-specific IgA and IgG level in *C. auris*-infected, *C. albicans*-infected and uninfected mice Representative flow plots and the absolute number of CD4+ IFNγ + T helper cells **(A, E)**, CD4+ IL5+ T helper cells **(B, F),** CD4+ IL17+ T helper cells **(C, G)** and CD4+ IL22+ T helper cells **(D, H)** from the large intestine of uninfected, *C. auris* infected and *C. albicans* infected are shown. Total IgA **(I)** and *Candida*-specific IgA **(J)** levels in the small intestine (SI), large intestine (LI) and serum from uninfected, *C. auris* and *C. albicans* infected mice were shown. Total IgG1 **(K)** and *Candida*-specific IgG1 **(L)** levels in the small intestine (SI), large intestine (LI) and serum from uninfected, *C. auris* and *C. albicans* infected mice were shown. For immune cell analysis, all groups had 8-10 mice, except for *C. albicans* infected mice for IL22+ immune cell analysis had 5 mice. For IgA and IgG1 determination, each group had 5-8 mice. Data represent mean ± SEM. For immune cells, statistical significance was calculated using the Kruskal-Wallis test followed by Dunn’s multiple comparison test. For IgA and IgG1 analysis, statistical significance was calculated using Mann-Whitney U test, with ns indicates non-significant and *p ≤ 0.05, **p ≤ 0.01 ***p ≤ 0.001 were considered significant.

T helper cells such as Th1, Th17 and Th22 cells play a critical role in antifungal defense in the intestine (31–36). Therefore, we examined the CD4 T helper cells in the intestine of uninfected, *C. auris* and *C. albicans* infected groups. The absolute number of CD4+ IFNγ+ T helper 1 cells were significantly increased in the *C. auris* infected group when compared to *C. albicans* infected group (**Figures 4A, E**). No difference in the absolute number of CD4+ IL5+ T helper 2 cells was observed among all the groups (**Figures 4B, F**). The absolute number of CD4+ IL17+ T helper 17 cells and CD4+ IL22+ T helper 22 were significantly increased in both *C. auris* and *C. albicans*-infected groups compared to uninfected groups (**Figures 4C, G** and **D, H**).

Next, we examined the B cell immune response in *C. auris* and *C. albicans* infected mice. Specifically, IgA and IgG1 play a critical role during *C. albicans* infection (19, 37). We examined the total and fungal specific IgA and IgG1 antibodies level in the intestinal contents and serum samples of uninfected, *C. auris* and *C. albicans* infected groups. Our results indicate that total IgA was not significantly altered in the gut contents and serum samples of uninfected, *C. auris* and *C. albicans* infected groups (**Figure 4I**). Fungus-specific IgA was not altered in the intestinal contents among all the groups (**Figure 4J**). However, *C. auris*-specific IgA was significantly increased in the serum samples of *C. auris*-infected mice compared to uninfected groups (**Figure 4J**). There was no change in *C. albicans-*specific IgA in the serum samples of *C. albicans*-infected mice compared to the uninfected group (**Figure 4J**). Total and fungal specific IgG1 were not significantly altered in the gut contents and serum samples of uninfected, *C. auris* and *C. albicans* infected groups (**Figures 4K, L**).

Collectively, Th1 cells were significantly increased in the intestine of *C. auris*-infected mice compared to the *C. albicans*-infected groups. The percentages of Th17 and Th22 cells were significantly increased in the intestine of both *C. auris* and *C. albicans* mice compared to uninfected mice. Serum samples of *C. auris* but not in the *C. albicans*-infected mice had a significant increase of *Candida* -specific IgA.

## Discussion

*C. auris* was recently identified as one of the major *Candida* species in patients all over the world. Major risk factors for invasive *C. auris* infections include solid organ transplantation, diabetes, chronic kidney disease and prior antibiotic use (4, 6, 38). The colonization and host immune response to *C. auris* in different host niches is still not clear. Recent evidence suggests that prior antibiotic therapy increases the susceptibility to *C. auris* infections (3–5). Previous findings from our lab and other groups indicate that antibiotic treatment increases the colonization and dissemination of *C. albicans* from the intestine of immunosuppressed mice (14, 16, 17). Similar to *C. albicans*, in this study we demonstrated that cefoperazone treatment increased the susceptibility to *C. auris* colonization and dissemination from the intestine. In a recent study, the colonization and dissemination potential of invasive and non-invasive strains of *C. auris* were compared using a cocktail of antibiotics (vancomycin, gentamicin, kanamycin, metronidazole and colistin) and cortisone acetate, steroidal immunosuppressive drug (39). Invasive strains showed higher colonization and dissemination from the intestine compared to non-invasive strains. In our study, we used South Asian clade of *C. auris* (CDC 0387) which is known to cause invasive infections in humans and mice (40). Collectively, these findings indicate that murine gut serves as a potential colonization site and invasive strains of *C. auris* can disseminate from the intestine of antibiotic-treated immunosuppressed hosts (39).

Antibiotic treatment alters gut microbiome composition which play a major role in fungal colonization in the intestine (14, 17, 41). Furthermore, *C. albicans* infection alters the microbiome composition of antibiotic-treated mice (17, 42). Our results indicate that relative abundance of firmicutes members mainly *Clostridiales* and *Paenibacillus* were considerably increased in the antibiotic-treated mice infected with *C. auris* compared to uninfected mice. However, previous findings suggest that antibiotic-treated mice infected with *C. albicans* increases the relative abundance of Proteobacteria members (17, 42). Unlike *C. albicans*, *C. auris* is not a commensal organism. The difference in the microbiome composition of antibiotic-treated infected with *C. albicans* and *C. auris* is a future area to investigate.

While the use of broad-spectrum antibiotics is a major predisposing risk factor for increased fungal colonization, dysregulation in the host defense system contributes to dissemination of *C. albicans* from the intestine (13–16). Recent evidence indicates that *C. auris* elicits less immune response compared to *C. albicans* (30). Therefore, we examined the mucosal immune response to *C. auris* and compared with *C. albicans* under antibiotic-treatment conditions. Surprisingly, the number of CD11b+ CX3CR1+ macrophages was significantly decreased in the intestine of *C. auris*-infected mice compared to *C. albicans* group. CD11b+ CX3CR1+ macrophages play a critical role in antifungal defense to *C. albicans* in the intestine (29). Tissue-resident macrophages is one of the key innate immune cells necessary to protect host from *Candida* infections (28). Macrophage depletion leads to decreased fungal clearance and higher mortality in mice (13). The reduction of CD11b+ CX3CR1+ macrophages in the intestine of *C. auris*-infected mice maybe an immune evasion strategy employed by this fungus. Previous finding indicates that *C. auris* (CDC 0387) persisted longer than *C. albicans* in the kidneys of infected mice (40). The authors speculated that the delayed clearance of *C. auris* was in part due to the differences in the innate immune recognition between *C. auris* and *C. albicans* (40, 43). In addition, alterations in intestinal microbiota can impair the turnover of CX3CR1+ macrophages in the intestine (44, 45). Future studies required to understand if microbiome alteration in the *C. auris* infected mice contribute to decreased number of CX3CR1+ phagocytes in the intestine. Furthermore, we observed a significant increase in fungal-specific IgA in the serum of *C. auris* but not in *C. albicans* infected mice compared to uninfected groups. A previous finding indicates that *C. albicans* but not *Saccharomyces cerevisiae* increased IgA in the small intestinal contents and serum samples of germ-free mice monocolonized with these fungi, respectively (19). In our model, conventional mice treated with cefoperazone antibiotic and infected with *C. auris* but not *C. albicans* increased fungus-specific IgA in serum. These results suggest that IgA response is species specific and may differ under germ-free and antibiotic-treated settings.

Taken together, broad-spectrum antibiotic increases the colonization and dissemination of *C. auris* from the intestine. Furthermore, our findings revealed that *C. auris* infection alters the microbiome composition in the antibiotic-treated mice. Differences in the mucosal immune response between *C. auris* and *C. albicans* infected mice were noticed under antibiotic-treated setting. Future studies on microbiota, metabolite and host interactions in the context of *C. auris* intestinal colonization is necessary to understand the pathogenesis of this emerging pathogen. Results from these studies will form a platform to develop novel microbiota and metabolite-based probiotic therapies to prevent and treat *C. auris* intestinal colonization and invasive infections in humans (46).

## Data availability statement

The raw data supporting the conclusions of this article will be made available by the authors, without undue reservation.

## Ethics statement

The animal study was reviewed and approved by Purdue University.

## Author contributions

DD performed the experiments, analyzed data, and prepared figures. HH reviewed flow analysis, reviewed, and edited the manuscript. ST designed the experiments, assisted in mice studies, and wrote the manuscript. All authors contributed and approved the final version of the manuscript.
